# The Interactive Effects of Education and Social Support on Blood Pressure in African Americans

**DOI:** 10.1093/geroni/igab046.3166

**Published:** 2021-12-17

**Authors:** DeAnnah Byrd, Yanping Jiang, Samuele Zilioli, Roland Thorpe, Peter Lichtenberg, Keith Whitfield

**Affiliations:** 1 Arizona State University, Phoenix, Arizona, United States; 2 Wayne State University, Detroit, Michigan, United States; 3 Wayne state university, Detroit, Michigan, United States; 4 Johns Hopkins Bloomberg School of Public Health, Baltimore, Maryland, United States; 5 University of Nevada Las Vegas, Las Vegas, Nevada, United States

## Abstract

This study examined whether the effects of received and provided social support on blood pressure (BP) would differ by education. Data from 602 African American adults (48-95 years) enrolled in the Baltimore Study of Black Aging—Patterns of Cognitive Aging were analyzed using multiple linear regression. We found no main effects of received and provided social support on BP. However, a significant moderation effect was observed for systolic BP, such that greater received social support was positively associated with higher systolic BP among individuals with low levels of education, adjusting for age, sex, chronic health conditions, and depressive symptoms. The findings demonstrate that social support and education have joint effects on blood pressure, which highlights the importance of considering psychosocial determinants of adverse cardiovascular health outcomes that disproportionately affect African Americans.

